# Antiprotozoal Drug Handling and Management Practices in Asella District, Central Oromia, Ethiopia

**DOI:** 10.1155/2021/6648328

**Published:** 2021-04-19

**Authors:** Ashenafi Ababu, Dereje Endashaw, Haben Fesseha, Mesfin Mathewos

**Affiliations:** ^**1**^ Friendship Poultry Enterprise, Addis Ababa, Ethiopia; ^**2**^ School of Veterinary Medicine, Wolaita Sodo University, P.O. Box 138, Wolaita Sodo, Ethiopia

## Abstract

**Results:**

The preliminary survey comprising 70 animal health professionals and animal owners was undertaken through observation, interview using a structured questionnaire, and discussion with key informants to evaluate their handling and management practices of antiprotozoal drugs. According to the finding of this preliminary survey, there was inadequate antiprotozoal drug distribution and only few drugs, namely, diminazene diaceturate, amprolium, and sulfa drugs, were available with different trade names being imported from different manufacturing countries. Among the current available antiprotozoal drugs circulating in the area, those manufactured and imported from China were highly distributed in the majority of veterinary drug shops. The assessment showed that there was a gap in achieving the required knowledge and practice of proper handling and management issues in the professionals and awareness problems in the community. The study indicated that there were inappropriate transportation and storage practices. Moreover, selling drugs without prescription, ignoring farmers without properly informing the drug withdrawal period, and administration of drugs by nonprofessionals were the other most critical and unethical practices in the area. It is emphasized that this inappropriate handling and management practice, scarcity of antiprotozoal drugs, easy accessibility, and distribution by nonprofessionals could potentially affect the quality, safety, and effectiveness of antiprotozoal drugs which may lead to drug resistance development.

**Conclusion:**

Hence, continuous awareness creation in the community, capacity building, training, and upgrading programs to the professionals, as well as strict enforcement of drug control and administration regulation of the country, are essential in the overall effort of improving animal health and productivity through the control of protozoan diseases in and around Asella.

## 1. Background

Protozoa are ubiquitous single-celled organisms, of which over 65,000 species have been described. Many occur as either free-living forms or harmless commensals, but their numbers include some of the most significant causes of disease in domesticated animals and humans [1]. Protozoal infections are common in tropical and subtropical countries where sanitary conditions, hygiene practices, and control of the vectors of transmission are inadequate. Two types of infections caused by the major types of protozoa of veterinary importance are the haemoparasitic such as *Trypanosoma*, *Babesia*, and *Theileria* and the common enteric *Coccidia*, *Toxoplasma*, and *Giardia* [2]. Among protozoan diseases caused by trypanosomes, dourine which is restricted to the Arsi-Bale Zone of Ethiopian highland areas has been recognized by local farmers for many years as “Lappessa” and found to be a threat to the life and productivity of the equine population [3].

Antiprotozoal drugs are used to treat infections caused by protozoa which either destroy or inhibit their growth and the ability to reproduce. Antiprotozoal drugs destroy or inhibit protozoa by interfering with metabolic processes, drugs interfering with reproduction and larval physiology and drugs interfering with neuromuscular physiology of parasites [4]. The control of trypanosomosis in Ethiopia relies on either curative or prophylactic treatment of animals with diminazene aceturate or isometamidium chloride [5], whereas the treatment of babesiosis is dependent upon the availability of a particular drug in the market. Although imidocarb is the first drug of choice [6], diminazene aceturate is widely used in the tropics [7].

Remedy for theileriosis is best observed by buparvaquone, which is a more effective and reliable drug than any other field conditions [8]. Diminazene aceturate is a currently available drug for the treatment of dourine, although not fully effective in curing [3]. But, it was found that Cymelarsan was quite effective in curing acutely or chronically infected horses at both 0.25 mg/kg and 0.5 mg/kg, and relapses were not observed up to a year after treatment [9, 10].

Some antibacterial and antihelmintic drugs also have antiprotozoal activity. *In vitro* activity of antiprotozoal drugs and monitoring of resistance is more difficult for antiprotozoal drugs because standardized susceptibility testing is not routinely performed for these pathogens. Besides, many antiprotozoal drugs are designed to be active in the lumen of the intestine for the treatment of intestinal protozoal infections, and the concentration of active drugs in the intestinal lumen after oral administration is difficult to measure. Therefore, the concentration of drug to which these pathogens are exposed is often not known. The activity and dosage regimens of antiprotozoal drugs are often based on the results of clinical trials, rather than concentration-exposure relationships between antiprotozoal drugs and the organism of interest [11].

Drug use is a complex subject involving the prescriber, the dispenser, the patient, and pharmaceutical institutions. It is influenced by factors such as drug availability, the prescriber's experience, knowledge of dispensers, and many more. Inappropriate drug use is a problem of the whole world; however, the degree of the problem is higher in developing countries like Ethiopia [12, 13]. Improper use of drugs may cause ineffective treatment and unnecessary wastage of resources and may harm the patient [14]. Medicinal products, and starting materials used in the manufacture of medicinal products, should be stored and transported under conditions that ensure that their quality is maintained. Manufacturers' recommendations concerning storage temperatures should be observed, and this may involve the use of specialized storage and transport facilities [15].

Even though the effectiveness of drugs is damaged due to problems such as lack of awareness on how to handle and manage the drugs, lack of understanding of the potential effects of drug misuse and abuse, and lack of required facilities, veterinary drug handling and management problems, illegal drug smuggling, and free of charge drug supply are widely occurring events in Ethiopia [4]. Therefore, the study was conducted to assess the current circulating antiprotozoal drugs, their handling, and management practice in and around Asella.

## 2. Methods

### 2.1. Study Area

The study was conducted in and around Asella Town of Arsi Zone, Oromia region, which is situated in central Ethiopia within 6°59′–8°49′ morth latitude and 38°41′–40°44′ east longitude in central Ethiopia at 175 km southeast of Addis Ababa. The area has an elevation ranging from 1780–3100 m.a.s.l and is characterized by mild subtropical weather ranging from 5 to 28°C. Livestock is a major agricultural resource in the area with 2,249,479 cattle, 1,395,824 small ruminants, 388,082 equines, and 11,716 camels [16]. The annual temperature range is 10 to 22.6°C. It has a daily maximum temperature that can reach up to 28°C and a minimum temperature of 10°C [17].

### 2.2. Study Subjects

The study subjects included in this study are government-owned veterinary clinics, private drug shops, animal health professionals, and animal owners (farmers) found in and around Asella.

### 2.3. Study Methods

A cross-sectional study was conducted from December 2017 to May 2018 to assess the current circulating antiprotozoal drugs, their handling, and management practice in Asella district, Oromia, Ethiopia. Briefly, the study was conducted by collecting data through participant observation, by interviewing professionals who work in veterinary clinics or private drug shops and animal owners/clients who come to the clinic for treatment of their animals or pharmacy to buy drugs using a structured questionnaire and discussion with key informants. The questionnaire was with the target of assessing the current management practice of antiprotozoal drugs used for prophylaxis and treatment of protozoal infections in the study area.

### 2.4. Sampling Methods

The study sites (government clinics and private pharmacies) were selected purposively due to ease of access and willingness to provide information. The total sample size considered during the questionnaire survey was 20 animal health professionals who were actively working with veterinary drugs, of which nine of them were working in private veterinary drug shops and the other eleven were veterinary pharmacy attendants who work in public veterinary clinics. Moreover, 50 animal owners or attendants coming for animal treatment in veterinary clinics and clients coming to a private pharmacy to buy drugs were also included.

### 2.5. Data Collection Methods

A total of 20 animal health professionals and 50 animal owners (farmers) were interviewed using a structured questionnaire to assess their knowledge, awareness, and practices towards safe handling and management of antiprotozoal drugs. A complete data-collecting format was prepared and used to collect data from each veterinary clinic and drug shop during close observation. During observation, key informant discussions with working professionals and some animal owners were also carried out.

A separate and structured questionnaire was prepared for the government veterinary clinicians, private drug shop owners, and attendants to assess the knowledge, attitude, and practice of the professionals towards veterinary drug handling and management practice issues. The relevant issues were incorporated in an open-ended form. Verbal consent was obtained from the respondents after the objective of the study was explained to them before starting the interview. The questionnaire was focused on antiprotozoal drug handling and management practices during acquisition, transportation, storage, dispensing, administration, and disposal of expired drugs.

### 2.6. Data Management and Statistical Analysis

The collected data were entered into Microsoft excel spreadsheet and summarized using STATA version 13 for descriptive statistics. The output of the analyzed data was presented and summarized using tabulation, charts, and frequencies (percentage) to describe the data.

## 3. Results

### 3.1. Assessment of Available Antiprotozoal Drugs in Asella District

Information was collected from four government-owned veterinary clinics and 9 private veterinary drug pharmacies located in Asella, Gonde, and Golgia during close visual observation. Accordingly, only three types of antiprotozoal drugs, namely, diminazene diaceturate, amprolium, and sulfa, were available in the area. These drugs are imported from different countries with different trade names given by their manufacturing company (Table 1).

Among the current available antiprotozoal drugs in the area, those manufactured and imported from China were highly distributed in the majority of veterinary drug pharmacies. Chinese-imported amprolium was found in 76.9%, diminazene diaceturate in 69.2%, and sulphadimidine sodium in 61.5% of the whole observed veterinary drug stores in the area (Table 2).

### 3.2. Sources of Antiprotozoal Drugs in and around Asella

**Figure 1:**
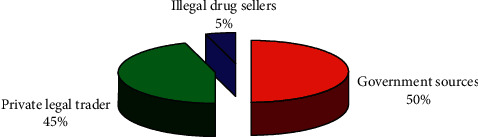
Sources of antiprotozoal drugs in and around Asella.

The questionnaire survey showed that the sources of antiprotozoal drug supply in Asella district were the governmental sources, private legal traders, and illegal drug sellers. According to the result of the questionnaire, above 50% of animal health professionals indicated that the source of antiprotozoal drugs was governmental, about 45% from the legal trader and the remaining 5% from illegal drug sellers (Figure 1). Also, the questionnaire survey from animal owners revealed that, out of 50 animal owner respondents, 7(14%) individuals were buying drugs from open markets supplied by illegal drug smugglers. However, these drug smugglers were not visually observed in the market during the study time.

### 3.3. Questionnaire Survey

#### 3.3.1. Knowledge and Practices of Animal Health Professionals

Out of the 20 respondents, in which 9 individuals were private veterinary drug dispensers or owners and 11 were government-owned veterinary clinic workers, 18 (90%) were male, 2 (10%) were female, and again, 12 (60%) of them were animal health assistants, 3 (15%) were animal health technicians, 2(10%) were animal science doctors, and 3 (15%) were veterinary doctors with 55% greater than five years, 35% between 1 and 5 year, and 10% less than a year of working experience.

The vast majority of the respondents (90%) use public vehicles to transport drugs, 85% do not store drugs according to the manufacturer's direction, and about 90% of the respondents sell drugs without prescription. Out of the 20 respondents, 18 (90%) professionals did not properly inform the drug withdrawal period to end users while giving treatment. The assessment indicated that 70% of the professionals had no enough knowledge of safe handling and management of drugs starting from acquisition to end users (Table 3).

#### 3.3.2. Awareness and Practice of Farmers

Out of 50 farmers interviewed, 58% had come to buy veterinary drugs and 42% to treat their animals. Of the total respondents, 62% replied as they do not have formal education. When their animals suffered from protozoan diseases, 64% of the farmers administer drugs bought from nearby veterinary pharmacies and 14% from open markets. It was noted that 62% of farmers replied as they were not satisfied with the response of therapy as the drug gives only some relief and 18% of them answered as the drug has no effect (Table 4).

### 3.4. Drug Administration Practices in the Area

**Figure 2:**
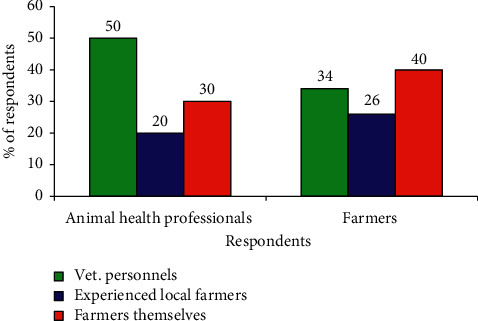
Antiprotozoal drug administration practices in the area.

The questionnaire survey of both animal health professionals and farmers revealed that 50% of professionals and 34% of farmers replied that drugs were administered by veterinary personnels, whereas 50% of professionals and 66% of farmers replied as drugs were administered by nonprofessionals (experienced local farmers and farmers themselves) (Figure 2).

## 4. Discussion

Since the livelihood of the community is mainly depending on mixed crop and livestock production, livestock is the major agricultural resource in the area [16]. The questionnaire survey and discussion with key informants indicated that there are different types of protozoan diseases in this area such as dourine, coccidiosis, and babesiosis which hinder the productivity of animals and cause economic loss to the community. In addition to this finding, it was reported that both animal owners and professionals interviewed in the area stated that dourine is a major health problem of horses causing high mortality and economic loss [10].

However, the present study disclosed that the presence of veterinary services and the amount of veterinary inputs, particularly antiprotozoal drugs, is not sufficient in the area because the assessment revealed only three types of antiprotozoal drugs, namely, diminazene diaceturate, amprolium, and sulfa drugs. These drugs are imported from different countries with different trade names. Among the current available antiprotozoal drugs in the area, those manufactured and imported from China were highly distributed in the majority of veterinary drug shops.

According to the result and discussion with key informants, diminazene diaceturate was one of the known and relatively highly purchased drugs by local farmers, which is mainly used to treat dourine-contracted horses. It can be indicated against several species of trypanosomes and piroplasms for cattle, camels, sheep, goats, and pigs. Although it is found with different trade names, DIMAZNC originated from China was distributed among 69.2% of the total drug stores assessed in the area. It was available in the form of powder (sachet) containing 2.36 grams of active hydrosoluble granules to be dissolved in 15 ml of water for one intramuscular injection at a dosage of 3.5 mg/kg (1 ml/20 kg). Diminazene diaceturate is not an effective treatment to cure clinical cases of dourine [18]. However, the absence of other qualified and curative therapeutic agents in the area against diseases such as dourine makes Diminazene diaceturate as a sole treatment option by animal health professionals and local farmers.

Amprolium was an anticoccidial drug used for therapeutic, prophylactic, and growth promotion, especially in poultry in the area. Chinese-imported amprolium present as AMPROLIUM 20% was found distributed among 76.9% of the total observed drug stores in the area. Amprolium imported from Jordan was also available as JOPROX^®^ (amprolium HCL, sulphaquinoxaline sodium, and vitamin K3) and JOPROL 20%^®^ (amprolium HCl). This drug is available in a powder form and can be given with drinking water for poultry and ruminants, mainly calves.

Sulfa drugs were broad-spectrum drugs that are used against bacteria and coccidian parasites. They were found in the form of sulphadimidine sodium imported from China and Trisulpha forte^®^ (sulphadimidine sodium, sulphadiazine sodium, and sulphathiazole sodium) imported from Jordan which were found distributed in 61.5% and 15% of the observed drug stores, respectively. Sulphadimidine sodium was found in 100 ml vial to be injected parenterally, whereas Trisulpha forte^®^ was found as a 100 gram powder form to be given in drinking water for 3 days at a dosage of 4-5 gram/50 kg for large animals and 5 gram/20 liter for poultry.

Governmental sources, private legal traders, and illegal drug sellers were the sources of antiprotozoal drug supply in the Asella district. The result of the questionnaire and discussion with key informants indicated that about 50% of antiprotozoal drugs were sourced from governmental drug stores, about 45% from private legal traders, and the remaining 5% from illegal drug sellers. The drugs from these sources reach end users through distribution via veterinary clinics and pharmacies as well as illegal black markets found in the area.

The governmental and private sources get the drugs from distributors in the capital city of the country, Addis Ababa, and other nearby cities such as Adama whereas illegal drug smugglers get the drugs from retailers without any legal license and from the black market inside and outside the country. Also, the questionnaire survey from animal owners revealed that, out of 50 respondents, 7(14%) were buying drugs from open markets supplied by illegal drug smugglers. Discussion with key informants showed that there are illegal drug smugglers who sell drugs in open markets and other shops in the area. However, these drug smugglers were not visually observed in the market during the study time.

Assessment of the knowledge, awareness, and practices of animal health professionals and farmers towards safe handling and management of antiprotozoal drugs using a structured questionnaire showed that there are many awareness problems and inappropriate practices. The result indicated 70% of the professionals had no enough knowledge and awareness on safe handling and management of drugs starting from acquisition to end user. All the professionals diagnose animals suffering from protozoan diseases tentatively and determine the dose of the drugs by estimating the animal's age and body weight. 80% of the professionals did not follow-up and complete the treatment of animals. On the other hand, 64% of the farmers administer drugs that were bought from nearby veterinary pharmacies and 14% from open markets when their animals suffered from protozoan diseases.

It is noted that public transport and storing drugs without referring the manufacturer's direction can expose the drugs to sunlight, unadjusted temperature, humidity, and other conditions including physical damage to the containers which disturb the stability of the drugs. However, the vast majority of the respondents (90%) use a public vehicle to transport drugs and 85% do not store drugs according to the manufacturer's directions. The other most critical and unethical practices in the area were selling drugs without the prescription, not informing the drug withdrawal period, and allowing nonprofessionals to administer drugs by themselves. However, 90% of the professionals were found selling drugs without a prescription and did not properly inform the drug's withdrawal period in which they did not advise end users about the potential effect of drug residue on public health. Moreover, the questionnaire survey revealed that 50% of professionals and 66% of farmers replied as drugs were administered by nonprofessionals (experienced local farmers and farmers themselves). But, 62% of the interviewed animal owners did not have formal education which indicated that they could not read and understand the information on the leaflets of the drugs and unable to get the dose rate and expiry date indicated on the leaflets. These conditions facilitate drug misuse and abuse, drug resistance, and unexpected outcome in both animal and human health.

The purpose, dosage, method of administration, contraindication, and other conditions of the drugs would not be clear to the sellers and users if they are not accompanied with a prescription. In [4], a discussion with the key informants showed that farmers buy drugs without prescription from pharmacies and also open markets easily and they put it in their house for a long period and have been practicing drug administration by themselves for treating their animals. Most of the farmers' treatment practice did not follow the proper dosage and site of injection, and they give underdosage of drugs on the basis to treat numerous animals at a time with the available amount of drug. and on the other hand, they give overdosage on the basis to relieve animal pain within a short period. They believe that all drugs are functional for a long period and can be used at any time.

Generally, the abovementioned conditions collectively showed that there were deep-rooted problems in the proper handling and management of veterinary drugs in general and antiprotozoal drugs in particular. This all is due to the knowledge gap in professionals and awareness problems in the community that can potentially affect the drug's quality, safety, and effectiveness. If the drugs are not handled and administered properly, there will be drug ineffectiveness that leads to high morbidity and mortality of animals that directly affects the life of the community [4]. The other potential threat of this improper drug handling and administration by nonprofessionals is development of drug resistance in which, finally, no drug will be left for treatment in the area because the variety of antiprotozoal drugs commonly used in the area are very few.

## 5. Conclusions

This study revealed that the presence of veterinary services and the amount of veterinary inputs, particularly antiprotozoal drugs, is not sufficient in the area. The result of this study disclosed the availability of only three types of antiprotozoal drugs, namely, diminazene diaceturate, amprolium, and sulfa drugs, which are imported from different countries with different trade names. Among the current available antiprotozoal drugs circulating in the area, those manufactured and imported from China were highly distributed in the majority of veterinary drug shops. The study showed that there were many inappropriate practices and attitudes associated with improper drug handling and management issues in the professionals and awareness problems in the community. This problem together with the easy accessibility of only few antiprotozoal drugs from private drug shops and also black markets could result in drug misuse and abuse which potentially affects the drug's quality, safety, and effectiveness. Generally, about 70% of the professionals had no enough knowledge of safe handling and management of drugs starting from acquisition to end user. This assessment showed that there is a knowledge gap on how to properly handle and manage veterinary drugs in general and antiprotozoal drugs in particular. This could be due to a lack of continuous awareness-creation training, limited capacity building and upgrading programs, and law enforcement of veterinary drug control and administration rules and regulations in the area. In conclusion, adequate variety and the number of antiprotozoal drugs from a known licensed drug manufacturing company should be supplied. Also, continuous awareness creation works to the community, capacity building, training, and upgrading educational level to the professionals should be done to reduce the present knowledge gap. Furthermore, strict enforcement of drug control and administration regulation of the country should be applied to avoid the abovementioned problems in the area.

## Figures and Tables

**Table 1:** Antiprotozoal drugs circulating in and around Asella.

Antiprotozoal drugs generic name	Drug's trade name and its manufacturing country				
Diminazene diaceturate	DIMAZNC (China)	INOMAZENE^®^ (France)	DIMINASAN (Holland)	DIMINAL^®^ (Korea)	RANGTRYPS (India)

Amprolium	AMPROLIUM 20% (China)	JOPROX^®^ and JOPROL 20%^®^ (Jordan)			

Sulfa drugs (sulphadimidine sodium)	YZ-SULFADIUM (China)	Trisulpha forte^®^ (Jordan)			

**Table 2:** Distribution of antiprotozoal drugs imported from different countries and their respective frequency of occurrence in the study area (*N* = 13).

Country of origin	Antiprotozoal drug trade names	Frequency N (%)
China	AMPROLIUM 20%	10 (76.9)
	DIMAZNC	9 (69.2)
	Sulphadimidine sodium	8 (61.5)
Jordan	JOPROX^®^	5 (38.5)
	JOPROL 20%^®^	3 (23)
	Trisulpha forte^®^	2 (15)
France	INOMAZENE^®^	3 (23)
Korea	DIMINAL^®^	1 (7)
India	RANGTRYPS	2 (15)
Holland	DIMINASAN	1 (7)

**Table 3:** Knowledge and practices of animal health professionals on safe handling and management of antiprotozoal drugs (*N* = 20).

Focused questionnaire items	Response category	Frequency (%)
Transportation of drugs	Public transport	18 (90)
	Special vehicle	2 (10)

Proper drug storage practice	Proper	3 (15)
	Improper	17 (85)

Selling drugs without prescription	Yes	18 (90)
	No	2 (10)

Diagnosis of protozoan diseases	Tentative	20 (100)
	Confirmatory	0 (0)

Dose determination using age and body weight	Yes	20 (100)
	No	0 (0)

Follow-up of treated animals	Yes	4 (20)
	No	16 (80)

Informing drug withdrawal periods to end users	Yes	2 (10)
	No	18 (90)

Knowledge of safe handling and management of drugs starting from acquisition to end users	Yes	6 (30)
	No	14 (70)

Disposal of expired drugs	Burning	17 (85)
	Not yet	3 (15)

**Table 4:** Awareness and practice of animal owners on the therapeutic management of antiprotozoal drugs.

Focused questionnaire items	Response category	Frequency (%)
Purpose of coming to the clinic/pharmacy	For treatment	21 (42)
	To buy drugs	29 (58)
Level of education	Primary	12 (24)
	Secondary	6 (12)
	College	1 (2)
	No formal education	31 (62)
Farmers practice when protozoan diseases exist in their animals	Buying and administration of veterinary drugs	32 (64)
	Traveling to the nearby veterinary clinic	18 (36)
Purchase of antiprotozoal drugs from the open market to treat animals	Yes	7 (14)
	No	43 (86)
Response to therapy	Cured	10 (20)
	Gives some relief	31 (62)
	No effect	9 (18)

## Data Availability

The datasets used and analyzed during the current study are available from the corresponding author on reasonable request.

## Supplementary Materials

Supplementary Materials Additional file- 1: Questionnaire format for antiprotozoal drug handling and management practices in Asella District, Ethiopia.

## References

[1] Taylor M. (2000). Protozoal disease in cattle and sheep. *In Practice*.

[2] Biobaku K., Takeet M., Olurode S., Oyewusi I., Oni O., Oloye A. (2010). The prevalence and clinico-haematological changes of protozoan diseases in food animals in Alabata, Abeokuta. *Nigerian Journal of Parasitology*.

[3] Yune N., Biratu G., Asefa G. (2017). Dourine (trypanosoma equiperdium infection): a review with special attention to Ethiopia. *European Journal of Biological Sciences*.

[4] Desta A. H. (2015). Veterinary drugs handling, management and supply chain assessment in Afar pastoral region of North East Ethiopia. *American Journal of Bioscience and Bioengineering*.

[5] Dagnachew S., Tsegaye B., Awukew A. (2017). Prevalence of bovine trypanosomosis and assessment of trypanocidal drug resistance in tsetse infested and non-tsetse infested areas of Northwest Ethiopia. *Parasite Epidemiology and Control*.

[6] Saad F., Khan K., Ali S., Akbar N. (2015). Zoonotic significance and prophylactic measure against babesiosis. *International Journal of Current Microbiology and Applied Sciences*.

[7] Demessie Y., Derso S. (2015). Tick borne hemoparasitic diseases of ruminants: a review. *Advances in Biological Research*.

[8] Nasir A. A. (2002). Effects of theileriosis on blood parameters of exotic cattle and efficacy of buparvaquone and oxytetracycline. *Pakistan Journal of Biological Sciences (Pakistan)*.

[9] Center for Food Security and Public Health (2009). *Equine Viral Arteritis*.

[10] Hagos A., Goddeeris B. M., Yilkal K. (2010). Efficacy of cymelarsan and diminasan against trypanosoma equiperdum infections in mice and horses. *Veterinary Parasitology*.

[11] Sykes J. E., Papich M. G. (2013). Antiprotozoal drugs. *Canine and Feline Infectious Diseases*.

[12] Sisay M., Mengistu G., Molla B., Amare F., Gabriel T. (2017). Evaluation of rational drug use based on world health organization core drug use indicators in selected public hospitals of eastern Ethiopia: a cross sectional study. *BMC Health Services Research*.

[13] Mensa M., Tadesse T., Ayele A. (2017). Assessment of drug use pattern by using world health organization core drug use indicators at public hospitals in Ethiopia. *Journal of Community Medicine and Health Education*.

[14] DACA (2006). *Standard Treatment Guidelines for Veterinary Practice*.

[15] Taylor J. (2001). Recommendations on the control and monitoring of storage and transportation temperatures of medicinal products. *The Pharmaceutical Journal*.

[16] APEDO (2007). *Socio Economic Data on Arsi Zone*.

[17] National Meteorological Services Agency (2007). *Climate Change National Adaptation Programme of Action (Napa) of Ethiopia*.

[18] Habte B., Bsrat A., Ashenafi H., Regassa F. (2015). Efficacy of some trypanocidal drug against Trypanosoma equiperdum OVI in experimentally infected mice in Debre Zeit, Ethiopia. *Jakarta Enterprise Beans*.

