# Supplementary material for: Antiprotozoal Drug Handling and Management Practices in Asella District, Central Oromia, Ethiopia
Source: Vet Med Int. 2021 Apr 19;2021:6648328. doi: 10.1155/2021/6648328 (PMC8075701; doi:10.1155/2021/6648328)
Supplement: Supplementary Materials — Additional file- 1: Questionnaire format for antiprotozoal drug handling and management practices in Asella District, Ethiopia. [file 6648328.f1.pdf]

Questionnaire format for Antiprotozoal Drugs Handling and Management Practices in Asella District, Central Oromia, Ethiopia

Part 1. General data collecting format during close observation and oral interview at each veterinary clinics and pharmacy in and around Asella

1. Date……………………………
2. Clinic ☐ pharmacy ☐
3. Name …………………………………………….
4. Address……………………………………………..
5. Phone no………………………………………….
6. Type of pharmacy A. government owned B. private
7. Available antiprotozoal drugs and their application (Table)

Drugs Name	Generic			
	Trade			
Country of origin				
Species of animals				
Dosage				
Route of administration				
Length of treatment				
Withdrawal period				
Mechanism of action				
Indications				

Part 2. Questionnaire for animal health professionals who actively working with veterinary drugs

General information

1. Address...Place of work...
2. Sex of respondent male ☐ female ☐
3. Status A. DVM B. AHA C. Animal science
   D, other(specify)………………………….
4. Work experience A. 1year B. 1-5 years C. >5years

Awareness and practices

5. Mention the common protozoan diseases in your area?
   …………………………………......…………………………………………………………
6. Mention common antiprotozoal drugs frequently purchased by clients?........................
   ………………………………………………………………………………………………
7. Do you have an acquisition form for drug transaction? Yes No
8. What do you register in the acquisition form?
   A. Batch number B. Quantity acquired C. Address of supplier
   D. other (specify)……………………………………….

9. From where do you get your drugs?
   A. government drug store B. legal private drug trader
   C. others (specify) ……………………………………..
10. How do you buy drugs to your shop?
   A. Face to face B. Through Agent
11. How do you transport drugs to your shop?
   A. Public transport B. Special vehicle
12. Do you store drugs in your shop according to manufacturer's direction? Yes No
13. Do you sell drugs without prescription? Yes No
14. Who is administering the drug?
   A. veterinary professionals B. experienced local farmers C. your self
15. Do you treat animals by yourself? Yes No
16. Do you have a case book? Yes No
17. How do you diagnose animals suffering from protozoal diseases?
   A. Tentative B. confirmatory
18. Which do you use to determine dose? A. age B. body weight C. both
19. Do you follow up and complete treatment of animals that you treat? Yes No
20. Do you advise end users on drug handling and administration? Yes No
21. Do you record and tell drug withdrawal periods to end users? Yes No
22. Do you refer drug's manufacturer direction like expiration date, leaflets and handling manuals? Yes No
23. Do you have enough knowledge on safe handling and management of drugs starting from acquisition to end user? Yes No
24. How do you dispose expired drugs? A. Burning B. Burying

Part 3: Questionnaire for animal owners targeting to assess the awareness and management practices of common antiprotozoal drugs

1. Address…………………………………..Kebele…………………………………..
2. Sex of respondent male ☐ female ☐
3. Level of education
   A. primary B. secondary C. college or above
   D. no formal education
4. Purpose of coming to clinic/pharmacy
   A. for treatment B. to buy drugs
5. Did anyone here tell you how to use drugs? Yes no
6. Did you understand what they explained to you? Yes no
7. Are you satisfied with the way your animal was treated? Yes no
8. Mention protozoan diseases you know in your area? ……………….................................
9. How do you combat protozoan diseases when it exists in your animals?
   A. Traditional medicine locally available

B. Buying and administration of veterinary drugs by their own

C. Travelling to nearby veterinary clinic

D. All alternatives are employed

10. Who administers the drugs?

A. Veterinary professionals B. experienced local farmers C. your self

11. Mention antiprotozoal drugs you know and experienced as a treatment for your animals?

Chemical name	Trade name	Local name

12. Do you buy antiprotozoal drugs from open market to treat your animals? Yes no

13. If yes, how do you prepare and administer to your animals?

A. Oral drenching B. injection

14. Does it cure the disease or give sort of relief?

A. cured B. give some relief C. no
